# A 19-Bit Small Absolute Matrix Encoder

**DOI:** 10.3390/s24051400

**Published:** 2024-02-22

**Authors:** Liming Geng, Guohua Cao, Chunmin Shang, Hongchang Ding

**Affiliations:** Mechanical Engineering Faculty, Changchun University of Science and Technology, Changchun 130022, China; gengjy511@163.com (L.G.); scm@cust.edu.cn (C.S.); dinghc@cust.edu.cn (H.D.)

**Keywords:** matrix encoder, matrix encoding, encoder miniaturization, code disk design

## Abstract

With the application of encoders in artificial intelligence and aerospace, the demand for the miniaturization and high measurement accuracy of encoders is increasing. To solve this problem, a new absolute matrix encoder is proposed in this paper, which can realize 19-bit matrix coding by engraving two circles of matrix code, and has the advantages of fewer circles of code disk engraving and higher measurement accuracy. This article mainly focuses on the design of a new matrix code disk, encoding and decoding methods, decoding circuit design, Matlab simulation analysis, and experimental error analysis. The experimental results show that the encoder designed in this paper achieves ultra-small volume Φ30 mm × 20 mm, and the angle measurement accuracy is 2.57”.

## 1. Introduction

A photoelectric encoder, also known as a photoelectric angle position sensor, is a digital angle measuring device that integrates light, machine and electricity. It is a sensor that converts the mechanical angular displacement into a pulse or digital quantity through photoelectric conversion [1,2,3]. Photoelectric encoders are widely used in many fields such as radar, photoelectric theodolite, CNC machine tool, robot and high-precision closed-loop speed control systems because of their high precision, fast response, non-contact measurement, anti-interference ability and stable performance [4,5].

With the development of aerospace and robotics technology in China, the miniaturization of absolute photoelectric encoders has been highly valued by enterprises and research institutes [6]. At present, the encoding methods for miniaturized absolute optoelectronic encoders mainly include Gray code, matrix code, single-ring Gray code, M-sequence code, and image encoding [7,8,9,10]. The matrix encoding method characterizes the encoding of different bits of traditional Gray codes on a circular code track, and its final output is the same as that of traditional Gray codes, but the number of code disk turns is reduced [11,12]. In 2010, Changchun Optical Machine Institute developed an eight-matrix absolute photoelectric encoder, which can realize 10-bit binary coding by engraving a two-circle matrix code track. In 2011, Changchun University of Science and Technology proposed a new matrix encoder solution, which realized 12-bit matrix coding by engraving two circles of matrix code; single-ring Gray code is a perfect encoding, but there are still many theoretical issues that need to be solved urgently [4,13]. Because the m-sequence code has no univariant, there are multi-bit changes between adjacent codes in use. In order to avoid coarse errors caused by code errors, the outer ring grating of the m-sequence code disk is the synchronous code track, and the inner ring is the m-sequence code [14,15]. When the encoder is designed with m-sequence code, multiple photosensitive probes need to be closely arranged. In order to realize the miniaturization of the encoder, an array charge-coupled device (CCD) is used to read the signal [16,17]. The introduction of an image sensor CCD increases the complexity of the light source and signal extraction, the resolution and accuracy of the measurement are more dependent on the quality of subsequent processing algorithms, and the complexity of the algorithms determines the response speed of the measurement device, ultimately restricting the maximum speed of the measured shaft [18,19,20].

By analyzing and summarizing the above encoding methods, the performance parameters of various encoders are compared in Table 1. The matrix Gray code encoding method of the optoelectronic encoder has the advantages of simple optomechanical structure, singularity, simple decoding, and high response rate compared to other encoding methods. However, there is a problem of insufficient accuracy in the case of a small number of code tracks.

This article proposes a new type of matrix encoder, which adopts a new method of code track engraving and encoding. The encoder only needs to engrave two circles of matrix code tracks to achieve 19-bit matrix encoding, achieving miniaturization of the encoder. Its volume is Φ30 mm × 20 mm, the encoder resolution reaches 19 bits, and the accuracy reaches 2.57”. At present, this encoder is a new type of encoder, which breaks through the limitations of traditional Gray codes and solves the contradiction between miniaturization and high measurement accuracy of encoders, meeting the requirements of aerospace, robotics, and nano-processing fields for encoders.

## 2. Working Principle

**Figure 1:**
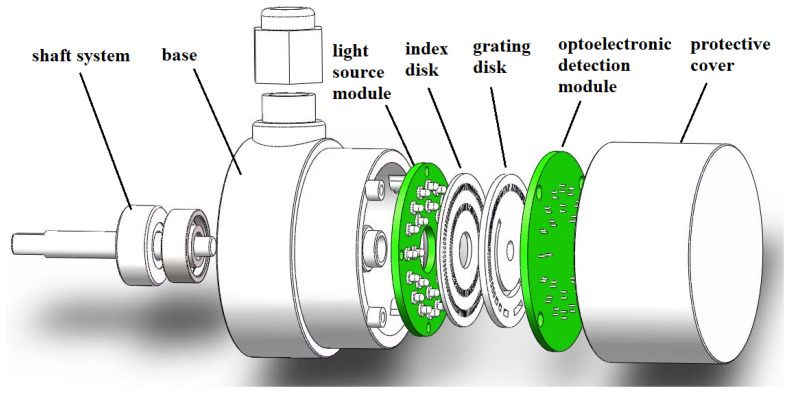
Explosion diagram of matrix encoder structure.

The exploded view of the matrix optoelectronic encoder proposed in this article is shown in Figure 1; it consists of a light source module, grating disk, index disk, optoelectronic detection module, shaft system, base, and protective cover. The light output by the LED light source module sequentially passes through the slit of the index disk and transparent area of the grating disk, and it is projected onto the opto-electronic receiver. The weak current signal output by the photoelectric receiver is amplified by a signal amplifier and a shaping circuit to output a square wave signal, and then decoded by a decoding circuit to output angle and position information.

As shown in Figure 1, when the encoder works, the grading disk rotates with the transmission shaft, and the LED light source module, photoelectric detection module and grading disk are stationary. Because of the obvious difference in the intensity of the optical signal received by the photoelectric detection module in the transparent area and the non-transparent area, we use binary codes “1” and “0” to represent the transparent area and the non-transparent area, respectively. By processing the binary code “1” and “0”, the decoding circuit converts the collected optical signal into an electrical signal that can be read by the machine for transmission and processing.

### 2.1. Design of Photoelectric Detection Circuit

Based on the advantages and disadvantages of different light sources and photodetectors, a selection was made. Considering that the encoder is an ultra small-volume encoder, Honeywell’s matching tubes, including SE2460 and SD2440, were selected in this paper to form the illumination and reception system. The main performance is shown in Table 2 and Table 3.

**Figure 2:**
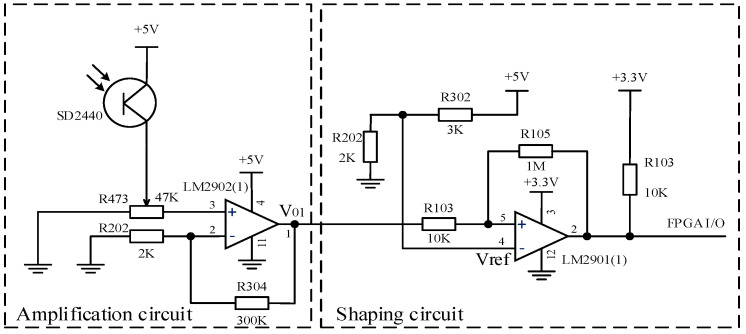
Detection circuit of phototransistor.

Due to the weak photoelectric current signal outputted by the photoelectric transistor, which cannot be directly read by the FPGA, we need to amplify and shape the current signal outputted by the photoelectric transistor to convert it into a square wave voltage signal. This article designs a photoelectric transistor amplification and shaping circuit as shown in Figure 2, which includes a voltage amplification circuit and a shaping circuit.

(1) Voltage amplification circuit

The voltage amplification circuit uses a low-power integrated operational amplifier, LM2902, which is connected through voltage series negative feedback. As shown in Figure 2, the gain of the negative feedback voltage amplification circuit is:

(1) $$A_{V}=\frac{V_{0}}{V_{i}}=\frac{A}{1+AF}$$

Among them, *A* represents the gain of the basic amplification circuit, *F* represents the feedback coefficient of the feedback network, *V_i_* represents the input voltage, *V*_0_ and represents the output voltage.

Because in the formula 1 + AF > >1, $A_{V}\approx \frac{1}{F}=150$.

(2) Shaping circuit

The shaping circuit converts the irregular voltage signal output by the voltage amplification circuit into a square wave. The voltage comparator compares the magnitude of two input voltages and outputs high and low levels based on the comparison results. The integrated voltage comparator chip LM2901 was selected, and positive feedback was introduced on the basis of the forward input single threshold voltage comparator to form a positive phase input hysteresis comparator with dual threshold values. It has strong anti-interference ability, and due to the acceleration of state transition by positive feedback, the edge of the output waveform is improved.

### 2.2. Design of Matrix Encoder

**Figure 3:**
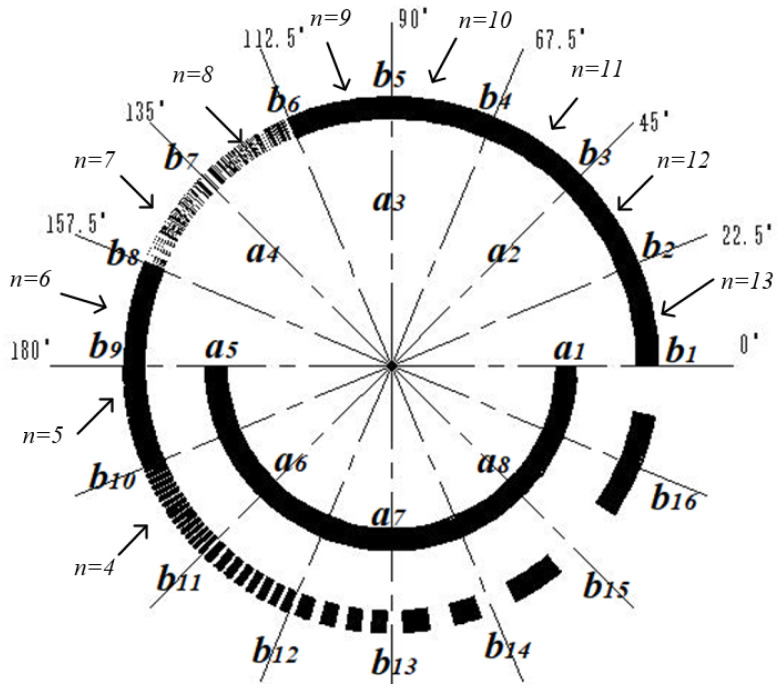
Code track distribution of matrix encoder.

**Figure 4:**
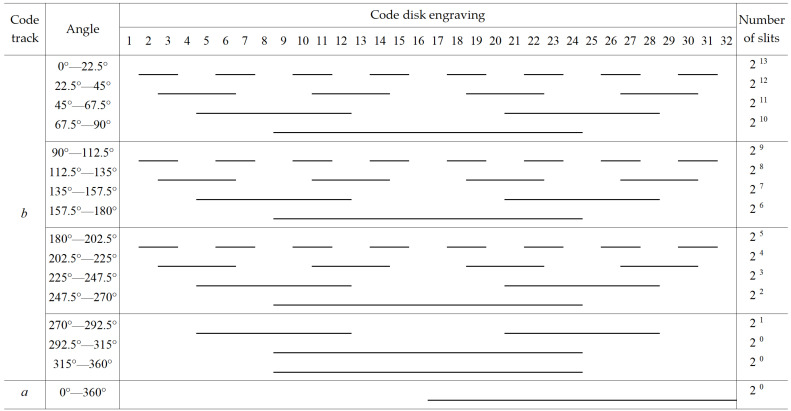
Planar expansion of matrix code tracks.

The traditional matrix encoder can read 8-bit binary codes by engraving three circles of matrix code, while the proposed 19-bit matrix encoder only needs to engrave two circles of code. As shown in Figure 3 and Figure 4, among them, 2^n^ is the number of lines in each sector code area, and *a*_1_*~a*_8_ and *b*_1_*~b*_16_ are used to indicate the installation positions of the *a* and *b* ring photodetectors, respectively. The design of the inner circle code channel is the same as that of the traditional Gray code channel. By increasing the number of light sources and detectors to eight pairs, 4-bit traditional Gray code information can be read. The outer circle code track is evenly divided into sixteen code regions by slits, and sixteen optical probes are evenly distributed around the circumference to read fifteen bits of traditional Gray code information. This encoding method reduces the number of code disk circles while increasing the number of encoder encoding bits to nineteen bits.

The specific code track design of the matrix encoder is as follows:

The inner circle, namely circle a, is provided with a code track, which is the same as the traditional code track, with a light-passing area within half a cycle of 180°~360°.

The outer circle, namely circle b, can be divided into 16 code areas, engraving the 5th~19th bit of the traditional code track, and the center angle of each code area is 22.5°. Area 0°~22.5° is the 1st code area, engraving the 19th bit of the traditional code track, engraving 2^13^ light-passing strips in the 22.5° circle range, called A_19_; 22.5°~45° is the 2nd code area, engraving the 18th bit of the traditional code track, engraving 2^12^ light-passing strips in the 22.5° circle range, called A_18_; 45°~67.5° is the 3rd code area, engraving the 17th bit of the traditional code track, engraving 2^11^ light-passing strips in the 22.5° circle range, called A_17_; 67.5°~90° is the 4th code area, engraving the 16th bit of the traditional code track, engraving 2^10^ light-passing strips in the 22.5° circle range, called A_16_; 90°~112.5° is the 5th code area, engraving the 15th bit of the traditional code track, engraving 2^9^ light-passing strips in the 22.5° circle range, called A_15_; 112.5°~135° is the 6th code area, engraving the 14th bit of the traditional code track, engraving 2^8^ light-passing strips in the 22.5° circle range, called A_14_; 135°~157.5° is the 7th code area, engraving the 13th bit of the traditional code track, engraving 2^7^ light-passing strips in the 22.5° circle range, called A_13_; 157.5°~180° is the 8th code area, engraving the 12th bit of the traditional code track, engraving 2^6^ light-passing strips in the 22.5° circle range, called A_12_; 180°~202.5° is the 9th code area, engraving the 11th bit of the traditional code track, engraving 2^5^ light-passing strips in the 22.5° circle range, called A_11_; 202.5°~225° is the 10th code area, engraving the 10th bit of the traditional code track, engraving 2^4^ light-passing strips in the 22.5° circle range, called A_10_; 225°~247.5° is the 11th code area, engraving the 9th bit of the traditional code track, engraving 2^3^ light-passing strips in the 22.5° circle range, called A_9_; 247.5°~270° is the 12th code area, engraving the 8th bit of the traditional code track, engraving 2^2^ light-passing strips in the 22.5° circle range, called A_8_; 270°~292.5° is the 13th code area, engraving the 7th bit of the traditional code track, engraving 2 light-passing strips in the 22.5° circle range, called A_7_; 292.5°~315° is the 14th code area, engraving the 6th bit of the traditional code track, engraving 1 light-passing strip in the 22.5° circle range, called A_6_; and 315°~360° is the 15th and 16th code area, engraving the 5th bit of the traditional code track, engraving 1 light-passing strip in the 45° circle range, called A_5_.

From the distribution of code tracks, we can easily see that:

(1) The circle a is exactly the same as the traditional Gray code track engraving method, which completes the encoding of 4-bit traditional code tracks through the placement of detectors.
(2) The method of engraving each code track in each code area of circle b is consistent with traditional code tracks, and the center angle of the adjacent detectors in circle b is the same as the center angle occupied by each code area. Therefore, when the physical code disk is turned to any position, 16 detectors read each of the 16 code areas, ensuring that there is no dead angle recording of the code disk information within 360 °.

### 2.3. Decoding Principles

The traditional encoder circuit consists of amplification, shaping, temporary storage, and display components. This system adds a matrix decoding module on the basis of the traditional decoding circuit. The traditional circuit part will not be elaborated here, and this article mainly introduces the principle of matrix decoding. The so-called matrix decoding is for converting the code tracks arranged in a matrix manner into the same code tracks as the conventional code tracks. In order to improve the processing speed of the circuit and ensure the real-time output of the angle, this paper adopts a hardware circuit based on FPGA for the design of the decoding system.

From Figure 3, it can be seen that eight photo-detectors are placed on the a-ring code track to convert the optical signal into high- and low-level signals. The placement method of the a-ring detector is as follows: Eight detectors are evenly distributed along the circumference, detector *a_1_* is placed at the 0° position, and *a_1_* to *a_8_* are placed counterclockwise in sequence.

**Figure 5:**
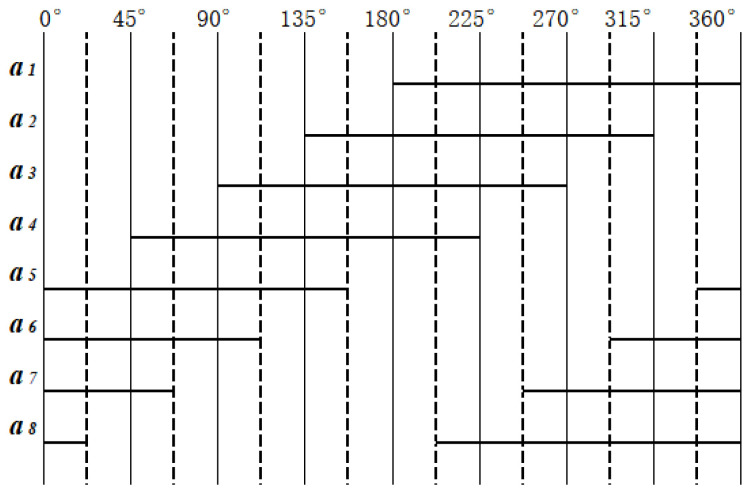
Changes of a-ring detector in one reading cycle.

The reading change of the a-ring detector within one cycle is shown in Figure 5; among them: the solid line represents high level, blank represents low level. The signals read by detectors *a*_1_ and *a*_3_, after amplification and shaping, are exactly the same as A_1_ and A_2_ encoded by traditional matrices; these two pulse signals serve as the first and second bit of the traditional Gray code. Therefore, the relationship between the eight signals *a_i_* in the first circle and the traditional Gray code *A_i_* is as follows:

(2) $$\left\{\begin{array}{l} A_{1}=a_{1}\\ A_{2}=a_{3}\\ A_{3}=a_{2}⊕a_{4}\\ A_{4}=a_{5}⊕a_{6}+a_{7}⊕a_{8} \end{array}\right.$$

On the b-ring code track, this paper proposes a scheme of sixteen quadrant matrix encoding, where sixteen detectors *b*_1_ to *b*_16_ are uniformly placed counterclockwise on a 360° circumference, with detector *b*_1_ placed at 0° and detector *b*_16_ placed at 337.5°.

When the encoder is turned to any position, each detector will read the light and dark stripes in different quadrants. When the encoder rotates clockwise, the relationship between the output pulse signal of each detector and the traditional Gray code is shown in Table 4. Taking the traditional Gray code *A_12_* as an example, detector *b*_1_ reads this bit information in the 8th code area, and detector b_2_ reads this bit information in the 7th code area.

The b-ring code track is divided into 16 code areas, and each code area number i corresponds to the 4-bit Gray code of the a-ring. The corresponding relationship is shown in Table 5.

The conversion relationship between the signal *b_i_* read by the b-ring detector and the traditional Gray codes *A*_5_*~A*_19_ is represented by matrix Equation (3), where *M_i_* represents the logical codes of code area *i*, such as: $M_{2}=\bar{A_{1}}\bar{A_{2}}\bar{A_{3}}A_{4}$.

(3) $$\left[\begin{array}{c} A_{5}\\ A_{6}\\ A_{7}\\ A_{8}\\ A_{9}\\ A_{10}\\ A_{11}\\ A_{12}\\ A_{13}\\ A_{14}\\ A_{15}\\ A_{16}\\ A_{17}\\ A_{18}\\ A_{19} \end{array}\right]=\left[\begin{array}{c} b_{15}\\ b_{14}\\ b_{13}\\ b_{12}\\ b_{11}\\ b_{10}\\ b_{9}\\ b_{8}\\ b_{7}\\ b_{6}\\ b_{5}\\ b_{4}\\ b_{3}\\ b_{2}\\ b_{1} \end{array}\begin{array}{c} b_{15}\\ b_{13}\\ b_{12}\\ b_{11}\\ b_{10}\\ b_{9}\\ b_{8}\\ b_{7}\\ b_{6}\\ b_{5}\\ b_{4}\\ b_{3}\\ b_{2}\\ b_{1}\\ b_{16} \end{array}\begin{array}{c} b_{13}\\ b_{12}\\ b_{11}\\ b_{10}\\ b_{9}\\ b_{8}\\ b_{7}\\ b_{6}\\ b_{5}\\ b_{4}\\ b_{3}\\ b_{2}\\ b_{1}\\ b_{15}\\ b_{16} \end{array}\begin{array}{c} b_{13}\\ b_{11}\\ b_{10}\\ b_{9}\\ b_{8}\\ b_{7}\\ b_{6}\\ b_{5}\\ b_{4}\\ b_{3}\\ b_{2}\\ b_{1}\\ b_{16}\\ b_{15}\\ b_{14} \end{array}\begin{array}{c} b_{11}\\ b_{10}\\ b_{9}\\ b_{8}\\ b_{7}\\ b_{6}\\ b_{5}\\ b_{4}\\ b_{3}\\ b_{2}\\ b_{1}\\ b_{16}\\ b_{15}\\ b_{14}\\ b_{13} \end{array}\begin{array}{c} b_{11}\\ b_{9}\\ b_{8}\\ b_{7}\\ b_{6}\\ b_{5}\\ b_{4}\\ b_{3}\\ b_{2}\\ b_{1}\\ b_{16}\\ b_{15}\\ b_{14}\\ b_{13}\\ b_{12} \end{array}\begin{array}{c} b_{9}\\ b_{8}\\ b_{7}\\ b_{6}\\ b_{5}\\ b_{4}\\ b_{3}\\ b_{2}\\ b_{1}\\ b_{16}\\ b_{15}\\ b_{14}\\ b_{13}\\ b_{12}\\ b_{11} \end{array}\begin{array}{c} b_{9}\\ b_{7}\\ b_{6}\\ b_{5}\\ b_{4}\\ b_{3}\\ b_{2}\\ b_{1}\\ b_{16}\\ b_{15}\\ b_{14}\\ b_{13}\\ b_{12}\\ b_{11}\\ b_{10} \end{array}\begin{array}{c} b_{7}\\ b_{6}\\ b_{5}\\ b_{4}\\ b_{3}\\ b_{2}\\ b_{1}\\ b_{16}\\ b_{15}\\ b_{14}\\ b_{13}\\ b_{12}\\ b_{11}\\ b_{10}\\ b_{9} \end{array}\begin{array}{c} b_{7}\\ b_{5}\\ b_{4}\\ b_{3}\\ b_{2}\\ b_{1}\\ b_{16}\\ b_{15}\\ b_{14}\\ b_{13}\\ b_{12}\\ b_{11}\\ b_{10}\\ b_{9}\\ b_{8} \end{array}\begin{array}{c} b_{5}\\ b_{4}\\ b_{3}\\ b_{2}\\ b_{1}\\ b_{16}\\ b_{15}\\ b_{14}\\ b_{13}\\ b_{12}\\ b_{11}\\ b_{10}\\ b_{9}\\ b_{8}\\ b_{7} \end{array}\begin{array}{c} b_{5}\\ b_{3}\\ b_{2}\\ b_{1}\\ b_{16}\\ b_{15}\\ b_{14}\\ b_{13}\\ b_{12}\\ b_{11}\\ b_{10}\\ b_{9}\\ b_{8}\\ b_{7}\\ b_{6} \end{array}\begin{array}{c} b_{3}\\ b_{2}\\ b_{1}\\ b_{16}\\ b_{15}\\ b_{14}\\ b_{13}\\ b_{12}\\ b_{11}\\ b_{10}\\ b_{9}\\ b_{8}\\ b_{7}\\ b_{6}\\ b_{5} \end{array}\begin{array}{c} b_{3}\\ b_{1}\\ b_{16}\\ b_{15}\\ b_{14}\\ b_{13}\\ b_{12}\\ b_{11}\\ b_{10}\\ b_{9}\\ b_{8}\\ b_{7}\\ b_{6}\\ b_{5}\\ b_{4} \end{array}\begin{array}{c} b_{1}\\ b_{16}\\ b_{15}\\ b_{14}\\ b_{13}\\ b_{12}\\ b_{11}\\ b_{10}\\ b_{9}\\ b_{8}\\ b_{7}\\ b_{6}\\ b_{5}\\ b_{4}\\ b_{3} \end{array}\begin{array}{c} b_{1}\\ B_{15}\\ b_{14}\\ b_{13}\\ b_{12}\\ b_{11}\\ b_{10}\\ b_{9}\\ b_{8}\\ b_{7}\\ b_{6}\\ b_{5}\\ b_{4}\\ b_{3}\\ b_{2} \end{array}\right]\cdot \left[\begin{array}{c} M_{1}\\ M_{2}\\ M_{3}\\ M_{4}\\ M_{5}\\ M_{6}\\ M_{7}\\ M_{8}\\ M_{9}\\ M_{10}\\ M_{11}\\ M_{12}\\ M_{13}\\ M_{14}\\ M_{15}\\ M_{16} \end{array}\right]$$

## 3. Simulation Analysis and Verification

**Figure 6:**
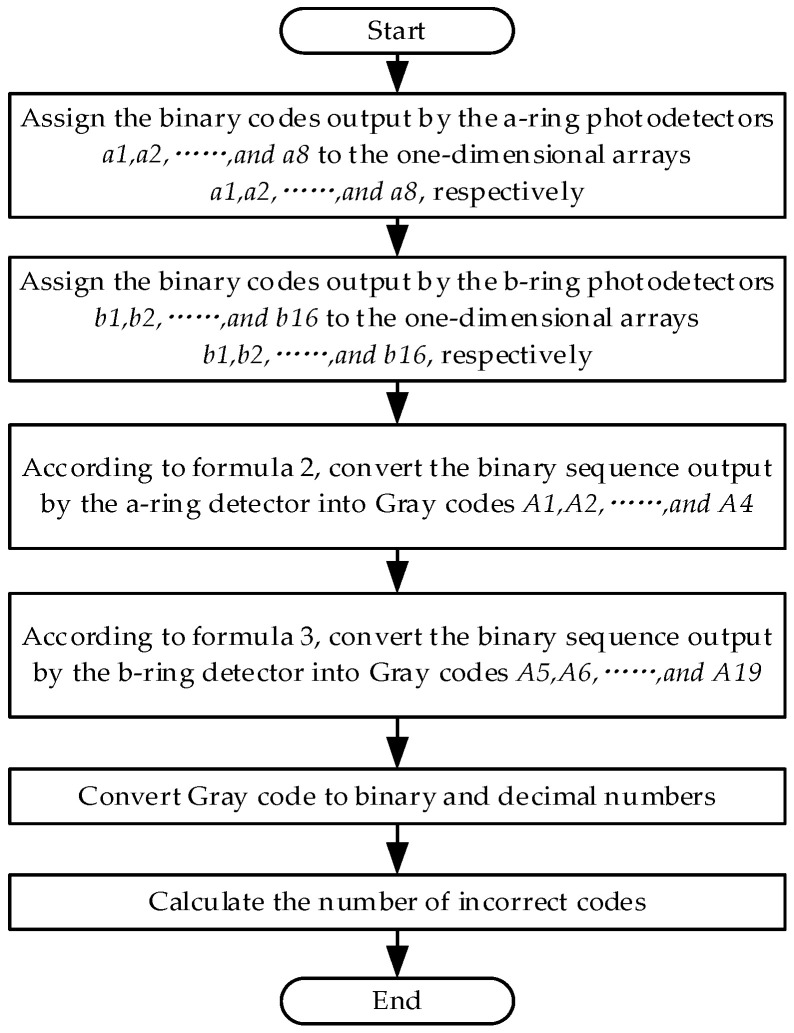
MATLAB simulation principle flowchart.

Based on the method of matrix encoder encoding and decoding, the decoding part of the encoder was simulated using MATLAB R2021a software, and the simulation principle is shown in Figure 6. The design idea is to assign the high- and low-level signals read by the eight photodetectors a_1_~a_8_ in the circle a to the one-dimensional arrays a_1_~a_8_. Similarly, assign the high- and low-level signals outputted by the 16 photodetectors in circle b to the one-dimensional arrays b_1_~b_16_. Use Formulas (2) and (3) to calculate A_1_~A_4_ and A_5_~A_19_, respectively. Finally, convert the Gray code into binary and decimal numbers, and calculate the number of errors. The simulation results prove that the number of error codes is zero, so the encoding and decoding methods used in this design are feasible.

In order to ensure the high-speed and real-time performance of the decoding circuit, this paper adopts a digital decoding circuit based on FPGA for high-speed decoding. The Gray code used in this paper is matrix encoded, and the entire decoding process can be divided into two parts: matrix Gray code → traditional Gray code → binary code.

(1) Matrix decoding

**Figure 7:**
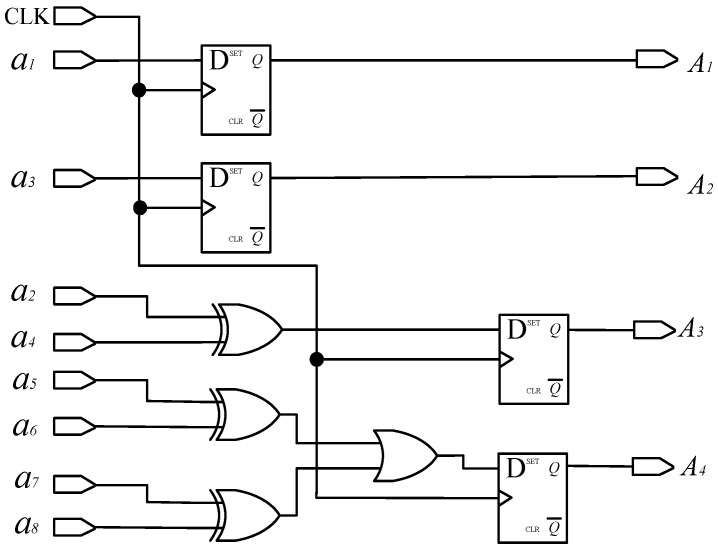
Decoding a-ring matrix code into a traditional Gray code circuit.

According to Formula (2), the decoding circuit for converting an a-ring code track to a traditional Gray code is shown in Figure 7, where the D flip-flop has two inputs, clock CLK and data D. And, the D flip-flop has two outputs: one is the main output, represented by Q, and the other is the reverse level of Q represented by $\bar{Q}$. When no clock input is applied to the D flip-flop or during the falling edge of the clock signal, the output Q remains unchanged from its previous value. If the clock signal is on the rising edge, the output Q remains consistent with the input D. CLK is the system clock, used to control the sampling frequency and synchronization of the system; *a*_1_, *a*_2_, …, and *a*_8_ are the level signals read by photo-detectors *a*_1_, *a*_2_,…, and *a*_8,_ respectively; and *A*_1_, *A*_2_, *A*_3_, and *A*_4_ represent the traditional Gray code.

**Figure 8:**
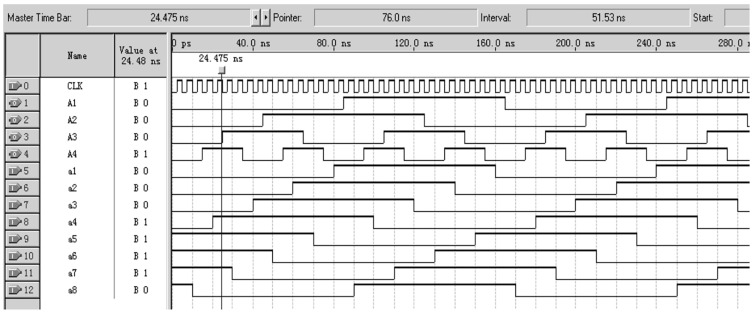
Simulation results of the decoding module for a-ring code track.

Due to the fact that the encoder designed in this article requires a large number of logical units and a fast running speed, Altera’s Cyclone series FPGA chip EP1C3T144C8 was selected, with the configuration chip EPC2. This FPGA chip has a dedicated global clock pin, and FPGA global clock resources are generally implemented using all copper layer technology. A dedicated clock buffer and driver structure were designed to allow the global clock to reach all configurable units inside the chip. The crystal oscillator selected is the small-sized programmable oscillator SIT8002UT chip from the SITIME company. Functional and timing simulations were conducted on the decoding module using Quartus II 9.0 software, and the results are shown in Figure 8. From the figure, it can be seen that the decoder starts working at the rising edge of the system clock CLK, and the simulation results are consistent with theoretical calculations. The decoding method and process of the b-ring code track are similar to the above. Due to the length of the article, this article only discusses the decoding implementation of the a-ring code track.

(2) Traditional Gray code → Natural binary code

**Figure 9:**
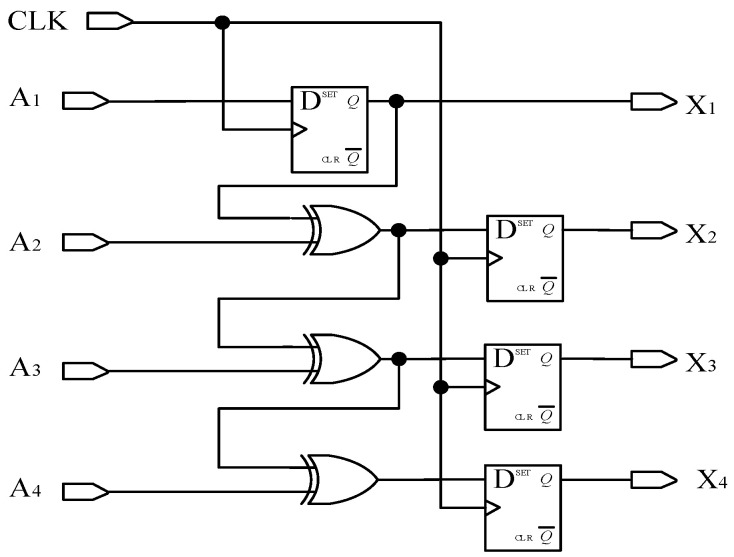
Schematic diagram of converting Gray code to binary code.

**Figure 10:**
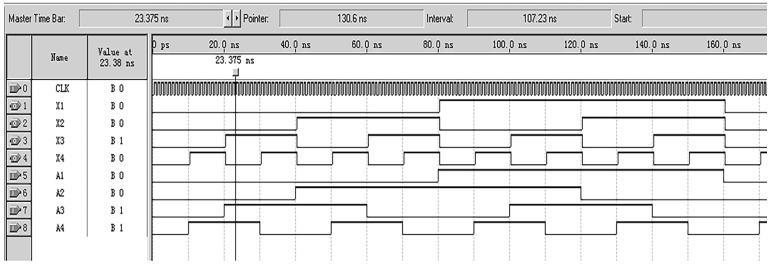
Simulation results of converting Gray code to binary code.

According to the rule of converting Gray code into natural binary code, the highest bit of Gray code is retained as the highest bit of binary code. According to the equation $X_{i}=A_{I}⊕X_{i-1}$, calculate the remaining bits of a binary number. This article designs a decoding circuit, as shown in Figure 9, and conducts functional simulation, as shown in Figure 10. The traditional Gray code is converted into commonly used natural binary codes, where A_1_, A_2_, A_3_, and A_4_ are traditional Gray codes, and X_1_, X_2_, X_3_, and X_4_ are natural binary codes.

## 4. Experimental Results and Error Analysis

**Figure 11:**
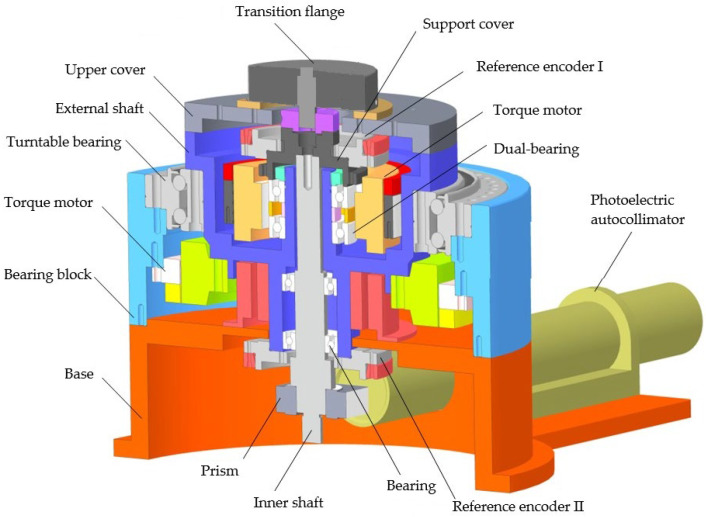
Three-dimensional model of optoelectronic encoder error detection platform.

**Figure 12:**
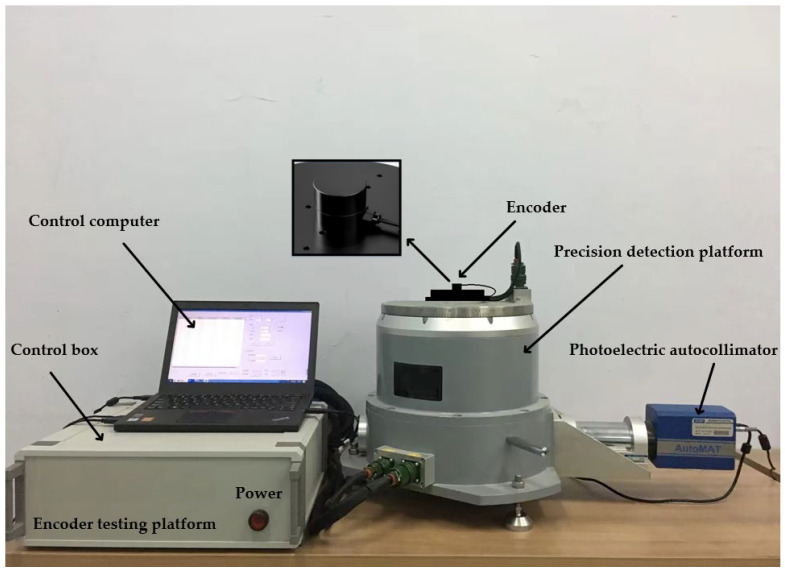
Optoelectronic encoder error detection platform.

On the basis of the above principles and simulation analysis, a prototype of the encoder was designed and manufactured, with geometric dimensions as follows: Φ30 mm × 20 mm, with a resolution of 19 bits. Using the direct comparison method for accuracy detection, precision detection is carried out using the direct comparison method, as shown in Figure 11 and Figure 12. An optical position closed-loop is formed using an autocollimator and a regular polyhedron as an angle reference. A fixed-position optical reflector is also installed to detect signal drift of the autocollimator during the process. The detection device consists of a fast detection platform and a control box, which can transmit the collected data to the host for display during the displayed time period. Compare the readings of the photoelectric encoder and detection equipment directly to calculate the instrument’s angle measurement error. The measurement method is shown in the following figure, and the detection data are shown in Table 6.

The performance of the testing equipment is ±360°continuous or compensatory testing, and it adopts an internal and external dual axis structure. The positioning accuracy of the inner and outer axis angular positions is ±0.2”, the stability of the angular positions is ±0.36”, the verticality and rotation accuracy of the axis system are ≤±2”, and the angular positioning resolution is ≤0.36”.

The standard deviation of its angle measurement error can be used represented as σ, and its standard deviation can be expressed as:

(4) $$\bar{x}=\frac{\sum_{i=1}^{n}x_{i}}{n},\;\delta_{i}=x_{i}-\bar{x},\;\sigma =\sqrt{\frac{{\left(x_{1}-\bar{x}\right)}^{2}+{\left(x_{2}-\bar{x}\right)}^{2}+\ldots \ldots +{\left(x_{n}-\bar{x}\right)}^{2}}{n}}=\sqrt{\frac{\sum_{i=1}^{n}\delta_{i}^{2}}{n}}$$

In the formula, *x_i_* is the measured value, $\delta_{i}$ is the random error, and $\bar{x}$ is the arithmetic mean of measured values.

According to Formula (4), it can be calculated that for the measurement error in both directions, the measurement accuracy of the photoelectric encoder is approximately 2.57”.

According to the sources of errors in the designer process of the encoders, encoder errors include theoretical errors, coding disc engraving errors and axis system errors, installation and debugging errors, etc. Among them, theoretical error is an inherent error in digital output devices, which is a fundamental error introduced during the conversion of analog signals to digital signals. The engraving error of the coding disk is caused by the engraving process, which introduces errors in the system when engraving the code track.

## 5. Conclusions

This article proposes a new type of encoder that achieves miniaturization, the matrix encoder and decoding circuit of the new encoder are designed and calculated, and simulation analysis is conducted on the designed encoder. The results show that the measurement accuracy can reach 2.57”(1σ). Compared with traditional research, the measurement accuracy has been significantly improved, which further demonstrates that the principles and methods proposed in this paper are practical and feasible. This is of great significance for improving the measurement accuracy of encoders and miniaturization.

The adoption of new encoding methods to reduce the size of the encoder disk and achieve the miniaturization of the encoder volume is an important development trend of photoelectric shaft angle encoders. The ultimate goal of absolute position coding research is to reduce the number of code tracks to one cycle, while also possessing the uniqueness and monotropy of traditional Gray codes. From this perspective, single-ring Gray codes are a perfect encoding. However, there are still many theoretical issues that urgently need to be addressed in the development of single-ring Gray code coding, which has become a new direction for its coding theory.

## Figures and Tables

**Table 1:** Comparison of performance parameters of rotary encoders based on different measurement methods.

Encoding Method	Number of Code Tracks	Resolution	Optical Characteristics			Monotropic	Algorithm Complexity	Response Speed
			Parallel Light	Focusing and Imaging	Detector			
Gray Code	*n*	360/2^n^	No	No	Phototransistor	Yes	Low	Fast
Matrix code	2	360/2^n^	No	No	Phototransistor	Yes	Medium	Medium
single-ring Gray code	1	Approaching 360/2^n^	Yes	Yes	CCD or CMOS	Yes	High	Slow
M-sequence code	2	360/2^n^	Yes	Yes	CCD or CMOS	No	High	Slow
image encoding	0	Subpixel, ang-ular second level	Yes	Yes	CCD or CMOS	No	High	Slow

**Table 2:** Performance parameters of light-emitting diode (LED) SE2460.

Model	Wavelength	Divergence Angle	Output Power		Voltage		Spectral Width	Spectral Temperature Drift
			Value	Test Conditions	Maximum Value	Test Conditions		
SE2460-003	935 nm	18°	1.0 mW	I_F_ = 50 mA	1.6V	I_F_ = 50 mA	50 nm	0.3 nm/°C

**Table 3:** Performance of phototransistor SD2440.

Model	Light Current		Receiving Angle	Rise and Fall Time	Dark Current	
	Value	Test Conditions			Value	Test Conditions
SD2440-003	1 mA	V_CE_ = 5 V Radiancy H = 20 mW/cm^2^	24°	15 μs	100 nA	V_CE_ = 5 V

**Table 4:** Relationship between b-ring signal and traditional Gray code.

	Code Area	1	2	3	4	5	6	7	8	9	10	11	12	13	14	15	16
Signal																	
b_1_		A_19_	A_18_	A_17_	A_16_	A_15_	A_14_	A_13_	A_12_	A_11_	A_10_	A_9_	A_8_	A_7_	A_6_	A_5_	A_5_
b_2_		A_18_	A_17_	A_16_	A_15_	A_14_	A_13_	A_12_	A_11_	A_10_	A_9_	A_8_	A_7_	A_6_	A_5_	A_5_	A_19_
b_3_		A_17_	A_16_	A_15_	A_14_	A_13_	A_12_	A_11_	A_10_	A_9_	A_8_	A_7_	A_6_	A_5_	A_5_	A_19_	A_18_
b_4_		A_16_	A_15_	A_14_	A_13_	A_12_	A_11_	A_10_	A_9_	A_8_	A_7_	A_6_	A_5_	A_5_	A_19_	A_18_	A_17_
b_5_		A_15_	A_14_	A_13_	A_12_	A_11_	A_10_	A_9_	A_8_	A_7_	A_6_	A_5_	A_5_	A_19_	A_18_	A_17_	A_16_
b_6_		A_14_	A_13_	A_12_	A_11_	A_10_	A_9_	A_8_	A_7_	A_6_	A_5_	A_5_	A_19_	A_18_	A_17_	A_16_	A_15_
b_7_		A_13_	A_12_	A_11_	A_10_	A_9_	A_8_	A_7_	A_6_	A_5_	A_5_	A_19_	A_18_	A_17_	A_16_	A_15_	A_14_
b_8_		A_12_	A_11_	A_10_	A_9_	A_8_	A_7_	A_6_	A_5_	A_5_	A_19_	A_18_	A_17_	A_16_	A_15_	A_14_	A_13_
b_9_		A_11_	A_10_	A_9_	A_8_	A_7_	A_6_	A_5_	A_5_	A_19_	A_18_	A_17_	A_16_	A_15_	A_14_	A_13_	A_12_
b_10_		A_10_	A_9_	A_8_	A_7_	A_6_	A_5_	A_5_	A_19_	A_18_	A_17_	A_16_	A_15_	A_14_	A_13_	A_12_	A_11_
b_11_		A_9_	A_8_	A_7_	A_6_	A_5_	A_5_	A_19_	A_18_	A_17_	A_16_	A_15_	A_14_	A_13_	A_12_	A_11_	A_10_
b_12_		A_8_	A_7_	A_6_	A_5_	A_5_	A_19_	A_18_	A_17_	A_16_	A_15_	A_14_	A_13_	A_12_	A_11_	A_10_	A_9_
b_13_		A_7_	A_6_	A_5_	A_5_	A_19_	A_18_	A_17_	A_16_	A_15_	A_14_	A_13_	A_12_	A_11_	A_10_	A_9_	A_8_
b_14_		A_6_	A_5_	A_5_	A_19_	A_18_	A_17_	A_16_	A_15_	A_14_	A_13_	A_12_	A_11_	A_10_	A_9_	A_8_	A_7_
b_15_		A_5_	A_5_	A_19_	A_18_	A_17_	A_16_	A_15_	A_14_	A_13_	A_12_	A_11_	A_10_	A_9_	A_8_	A_7_	A_6_
b_16_		A_5_	A_19_	A_18_	A_17_	A_16_	A_15_	A_14_	A_13_	A_12_	A_11_	A_10_	A_9_	A_8_	A_7_	A_6_	A_5_

**Table 5:** Correspondence between code area and logic code.

Code Area *i*	1	2	3	4	5	6
Gray code	B0000	B0001	B0011	B0010	B0110	B0111
Logic code *M_i_*	$\bar{A_{1}}\bar{A_{2}}\bar{A_{3}}\bar{A_{4}}$	$\bar{A_{1}}\bar{A_{2}}\bar{A_{3}}A_{4}$	$\bar{A_{1}}\bar{A_{2}}A_{3}A_{4}$	$\bar{A_{1}}\bar{A_{2}}A_{3}\bar{A_{4}}$	$\bar{A_{1}}A_{2}A_{3}\bar{A_{4}}$	$\bar{A_{1}}A_{2}A_{3}A_{4}$
Code area *i*	7	8	9	10	11	12
Gray code	B0101	B0100	B1100	B1101	B1111	B1110
Logic code *M_i_*	$\bar{A_{1}}A_{2}\bar{A_{3}}A_{4}$	$\bar{A_{1}}A_{2}\bar{A_{3}}\bar{A_{4}}$	$A_{1}A_{2}\bar{A_{3}}\bar{A_{4}}$	$A_{1}A_{2}\bar{A_{3}}A_{4}$	$A_{1}A_{2}A_{3}A_{4}$	$A_{1}A_{2}A_{3}\bar{A_{4}}$
Code area *i*	13	14	15	16		
Gray code	B1010	B1011	B1001	B1000		
Logic code *M_i_*	$A_{1}\bar{A_{2}}A_{3}\bar{A_{4}}$	$A_{1}\bar{A_{2}}A_{3}A_{4}$	$A_{1}\bar{A_{2}}\bar{A_{3}}A_{4}$	$A_{1}\bar{A_{2}}\bar{A_{3}}\bar{A_{4}}$		

**Table 6:** Detection data of matrix encoder.

Angle	0°	15°	30°	45°	60°	75°	90°	105°
Measuring error (Prograde)	0”	2.5”	3.1”	−2.4”	2.3”	−1.2”	2.3”	3.4”
Measuring error (reversal)	0”	3.2”	2.6”	−2.5”	−1.8	3.1”	−2.6”	3.7”
Angle	120°	135°	150°	165°	180°	195°	210°	225°
Measuring error (Prograde)	2.2”	−1.9”	1.6”	2.5”	−1.8”	2.1”	3.5”	−2.4”
Measuring error (reversal)	2”	2.4”	−3.5”	1.9”	−2.2”	1.8”	2.3”	−1.6”
Angle	240°	255°	270°	285°	300°	315°	330°	345°
Measuring error (Prograde)	2.6”	3.1”	−2.6”	2.7”	3.9”	−2.6”	3.1”	2.2”
Measuring error (reversal)	−2.1”	3.2”	−2.8”	3.4”	3.2”	2.6”	−1.2”	3.3”

## Data Availability

Data are contained within the article.
